# Evaluation of the Compliance, Acceptance, and Usability of a Web-Based eHealth Intervention for Parents of Children With Infantile Hemangiomas: Usability Study

**DOI:** 10.2196/resprot.2897

**Published:** 2013-12-17

**Authors:** Marlies de Graaf, Joan Totte, Corstiaan Breugem, Harmieke van Os-Medendorp, Suzanne Pasmans

**Affiliations:** ^1^Wilhelmina Children’s HospitalDepartment of Pediatric Dermatology and AllergologyUniversity Medical Center UtrechtUtrechtNetherlands; ^2^University Medical CenterDepartment of Dermatology and AllergologyUniversity Medical Center UtrechtUtrechtNetherlands; ^3^Children’s Hospital Erasmus University Medical Center-SophiaDepartment of Pediatric DermatologyErasmus University Medical CenterRotterdamNetherlands; ^4^Wilhelmina Children’s HospitalDepartment of Pediatric Plastic SurgeryUniversity Medical Center UtrechtUtrechtNetherlands

**Keywords:** eHealth, e-learning, Internet, compliance, acceptance, usability, dermatology, optimizing care, infantile hemangioma, child

## Abstract

**Background:**

Infantile hemangiomas (IH) are common benign vascular tumors in children. Recognition and timely referral of high risk IH to specialized centers is important. This might be achieved by involving parents in the care for IH by means of an eHealth intervention.

**Objective:**

The objective of our study was to evaluate parent compliance, acceptance, and usability of an open access, Web-based eHealth intervention (including e-learning and e-consult) designed to increase parents’ knowledge and (risk) evaluation of IH.

**Methods:**

A cross-sectional study of parents who completed the eHealth intervention between October 2010 and November 2012 was carried out. All parents were sent a study questionnaire. Questions to evaluate compliance (to the advice given by a dermatologist during e-consultation) were asked. Acceptance and usability were evaluated by using the modified Technology Acceptance Model.

**Results:**

A total of 224 parents completed the eHealth intervention and received the questionnaire, 135/224 parents responded (response rate was 60.3%). There were 128/135 questionnaires that were completed and included. A total of 110/128 (85.9%) parents were compliant to the advice of the dermatologist. There were 116.8/128 (91.3%) that perceived the eHealth intervention as useful and almost all parents (98.4%, 126/128) found the information in the e-learning clear. There were 29/128 (22.7%) that experienced technical problems. The majority of the parents (94.5%, 121/128) found the eHealth intervention reliable and most of them (98.4%, 126/128) would recommend the eHealth intervention to other parents. Noncompliant parents judged the eHealth intervention significantly less reliable compared to compliant parents (71%, 10/14 versus 97.3%, 107/110; *P*=.003).

**Conclusions:**

Parents of children with an IH showed a high compliance (85.9%, 110/128) to the advice of the dermatologist given via our Web-based eHealth intervention. This high compliance might be positively influenced by the good acceptance and usability of the eHealth intervention and might result in timely presentation and treatment of children with high risk IH in specialized centers.

## Introduction

### Infantile Hemangiomas

Infantile hemangiomas (IH) are common benign vascular tumors with a unique growth pattern [1-3]. Although most IH have an uncomplicated course, 24% of the patients experience complications, such as ulceration, bleeding, functional impairment, life-threatening risk, or cosmetic risk of which 38% need treatment [4]. Also, a segmental IH can be associated with congenital malformations and requires diagnostic evaluation [4]. Currently, complicated IH can be treated with beta blockers, like propranolol [5,6]. Correct initial diagnosis and timely referral of patients at risk of complications is important since early intervention may prevent complications [4,7].

### Parents and E-Learning

In order to ensure timely referral of high-risk IH, it is imperative for parents and health care professionals to have knowledge about IH and risk factors for developing complications. e-learning is widely used to increase knowledge, including the field of dermatology [8-14]. Parents use the Internet as an information source for the disease of their child, and the use of an educational e-learning module to increase patients’ knowledge has also been reported [15-18].

To increase parents’ knowledge about IH and its complications, we have developed an open access Web-based eHealth intervention [19,20]. This eHealth intervention consisted of an e-learning module and an e-consult (including a teledermatology consultation). Advice on diagnosis, risk of complications, and need to be seen by a medical specialist was given. If parents follow this advice (compliance to the advice) it might contribute to timely referral of high-risk patients to a medical specialist.

### Parent Compliance

Patient/parent-compliance (“the extent to which the parent’s behavior coincides with the advice of the dermatologist”) is essential for the success of this eHealth intervention. Compliance to medication has been extensively described in the literature. However, little is known about compliance to advice given via eHealth.

The goal of this study was to evaluate the compliance of the parents to the advice given by the dermatologist via the e-consult. Second, the acceptance and usability of this eHealth intervention were determined.

## Methods

### Design and Participants

**Figure 1:**
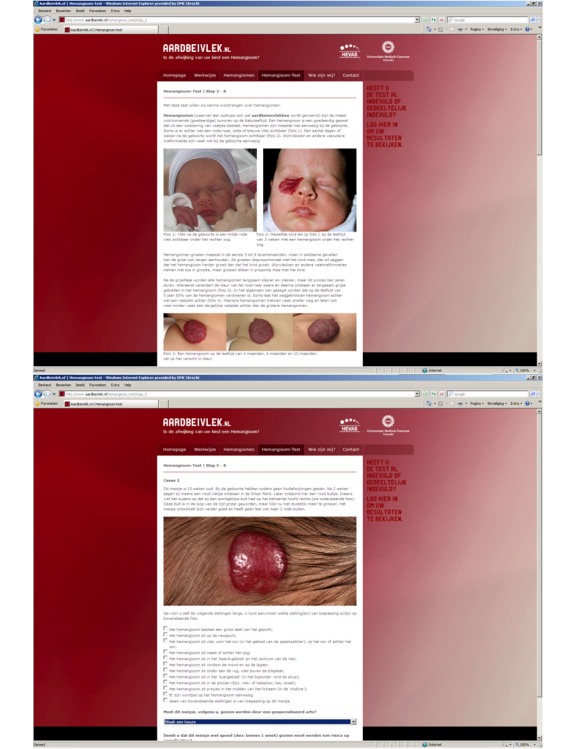
Illustrative screenshots of the e-learning module (in Dutch). The top image shows general information about infantile hemangiomas. The bottom image shows a case scenario of an infantile hemangioma on the scalp (Case 2).

A cross-sectional study was carried out after participation in the open access Web-based eHealth intervention [19], consisting of an e-learning module and e-consult (Figure 1 shows illustrative screenshots).

The Dutch patient support group for Hemangiomas and Vascular Anomalies (HEVAS) and the University Medical Center Utrecht (UMCU) supported the eHealth intervention, and their logos were displayed on the home page. Parents were referred to the eHealth intervention by a link on the home page of HEVAS [21], by their child’s youth or primary health care provider, or by surfing the Internet. Participation was voluntary and free of charge.

After registration on the website, parents received a password to start the e-learning module and e-consult. By using a password, safe uploading of personal information on the website was guaranteed. During the e-learning module parents were informed about IH and its complications and two illustrative cases were presented. During the e-consult parents were asked to provide one photograph of the skin lesion of their child and to give information regarding its growth pattern. A dermatologist of the Center for Congenital Vascular Anomalies Utrecht (CAVU) judged this photograph. In case the dermatologist was unable to make a proper diagnosis, due to lack of quality of the photograph, parents were asked for a new photograph or referred to their general practitioner (GP). Advice on diagnosis, risk of complications, and need to be seen by a medical specialist was given within 5 working days by email [20]. Parents were advised whether or not to go to a medical specialist and whether there was urgency. All parents of a child with a suspected IH, who fully went through the e-learning and e-consult between October 2010 and November 2012, were eligible for study participation and received a study questionnaire by email. The time between participation in the eHealth intervention and completing the questionnaire was variable. Demographic information of the parents was obtained. The ethics committee of the University Medical Center Utrecht approved the study.

### Theoretical Framework and Study Questionnaire

#### Compliance, Acceptance, and Usability

A questionnaire was developed to evaluate the variables–compliance, acceptance, and usability of the eHealth intervention.

#### Compliance

Compliance was defined as the extent to which the parent’s behavior coincides with the advice of the dermatologist. By means of the e-consult, parents were given an advice about the diagnosis of the skin lesion of their child (IH/no IH/uncertain) and about the need to visit a medical specialist (no need/need/urgent need). In case of “need to visit a specialist,” parents were first referred to their GP because in the Netherlands a referral of the GP is required for visiting a medical specialist. In case of “no need to visit a specialist,” parents were only advised to go to their GP if the IH was growing rapidly or became ulcerated. In all cases of “no IH” or “uncertain diagnosis” (in which the dermatologist was unable to diagnose the skin abnormality using the provided information by the parents), parents were advised to go to their GP. In order to determine the compliance, questions regarding visits to GP/medical specialists, additional diagnostics, and initiated treatment were asked. The time between the advice and the actual appointment with a specialist was also evaluated by asking the parents.

#### Acceptance and Usability

The acceptance and usability of the eHealth intervention were evaluated by using a modified Technology Acceptance Model (TAM). The TAM is the most widely applied model to describe consumer acceptability [22,23]. Technology acceptance is defined as “an individual’s psychological state with regard to his or her voluntary or intended use of a particular technology” [24]. The TAM theorizes that an individual’s behavioral intention to use a technology is determined by two beliefs: (1) perceived usefulness (PU) and (2) perceived ease of use (PEU) [25]. It has proved to be suitable for different genders, age groups, cultures, levels of information technology competency, and in both obligatory and voluntary usage settings [26]. Health care professionals have tested the TAM for the prediction of adoption of telemedicine, and its reliability, robustness, and validity have been demonstrated [26-28]. To determine the acceptance and usability of our eHealth intervention, we have modified the TAM based on the Chau and Hu’s model of telemedicine acceptance [29]. We have added the dimension “attitude towards use” to the original TAM, because behavioral intention is also determined by attitude, which is influenced by PU and PEU [29,30]. Attitude can be defined as “the perception by an individual of the positive or negative consequences related to adopting the technology.” Questions to evaluate acceptance and usability were developed following the modified TAM.

#### Study Questionnaire

The study questionnaire consisted of 24 questions, grouped into three variables (demographic information, compliance, acceptance and usability) (Table 1). Acceptance and usability was subdivided using the three dimensions of the TAM (PU, PEU, and attitude). There were 12 questions that were rated on a three-point scale (agree, no agreement/no disagreement, disagree). There were 7 questions that could be answered with “yes” or “no,” and with the final question parents were asked to rate the eHealth intervention (including e-learning and e-consult) on a 0-10 scale (0=very bad, 10=excellent). At the end of the questionnaire there was an open field for comments and suggestions.

**Table 1:** Questions used to evaluate compliance, acceptance, and usability.

Variable	Dimension	Related questions	Example
Demographic information		1-4	Gender, age, relation to the patient, and education level
Compliance to advice		5-15	Did you visit your general practitioner after our advice?
Acceptance and usability	Perceived usefulness	16a-16e, 19a-19d	The e-learning module is useful to determine if my child is at risk for complications
	Perceived ease of use	17, 20, 21a-21d, 23	The information of the e-learning is understandable
	Attitude	8, 18, 22, 24	I would recommend the e-learning module to other people

### Analyses

Only fully completed questionnaires were used for evaluation. Descriptive analyses were used to evaluate the compliance, acceptance, and usability.

Fisher’s exact tests were used to evaluate the difference in acceptance, usability, and attitude between compliant parents and noncompliant parents.

## Results

### The Parent Questionnaire

A total of 224 parents completed the eHealth intervention and received the questionnaire, 135/224 parents responded (response rate, 60.3%). There were 128/135 questionnaires completed and included in this study. Reasons for not responding on the questionnaire are unknown. Parent characteristics are shown in Table 2.

**Table 2:** Characteristics of the parents (N=128).

Characteristic		Frequency, n (%)
**Gender**		
	Men	10 (7.8)
	Women	118 (92.2)
**Age**		
	< 20 years	0 (0)
	20-29 years	20 (15.6)
	30-39 years	85 (66.4)
	> 40 years	21 (16.4)
	Unknown	2 (1.6)
**Relation to the child**		
	Parent	127 (99.2)
	Caretaker (grandparent)	1 (0.8)
**Highest educational level**		
	Low	6 (4.7)
	Moderate	32 (25.0)
	High	88 (68.8)
	Unknown	2 (1.6)
**Previously received information** ^a^		
	None	4 (3.1)
	Internet	66 (51.6)
	Primary health care provider	58 (45.3)
	Specialist	6 (4.7)
	Unknown	32 (25.0)

^a^Some parents previously received information from multiple sources.

### Parent Compliance With Medical Advice

**Figure 2:**
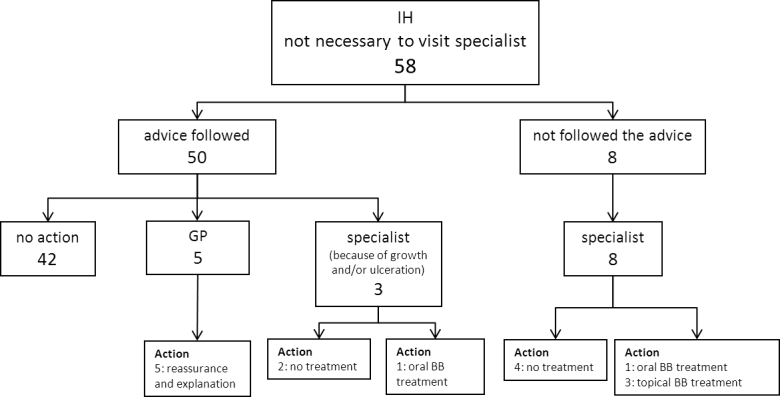
Flowchart of the compliance of the parents who were advised not to visit a medical specialist by the dermatologist via e-consultation. The flowchart shows which doctor the parents visited and to what actions (eg, diagnostic evaluation and/or treatment) it has led. The figures indicate the number of patients. Infantile hemangioma(s) (IH); general practitioner (GP); and beta blocker (BB).

**Figure 3:**
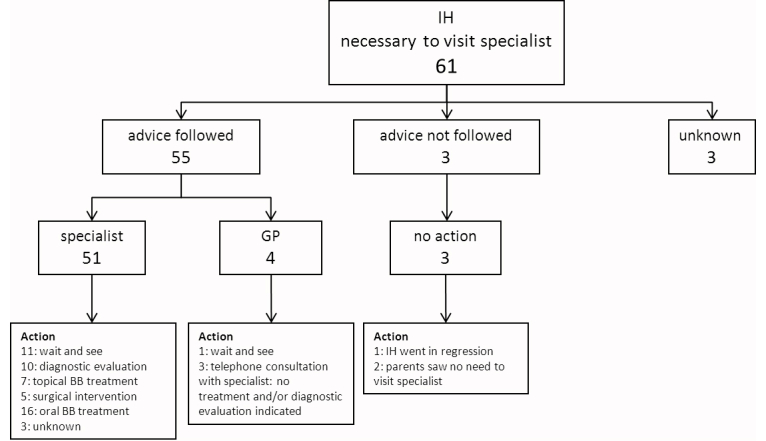
Flowchart of the compliance of the parents who were advised to visit a medical specialist by the dermatologist via e-consultation. The flowchart shows which doctor the parents visited and to what actions (eg, diagnostic evaluation and/or treatment) it has led. The figures indicate the number of patients. One patient, who followed the advice of the dermatologist and went to a specialist, underwent both diagnostic evaluation and topical beta blocker treatment was initiated. Infantile hemangioma(s) (IH); general practitioner (GP); and beta blocker (BB).

**Figure 4:**
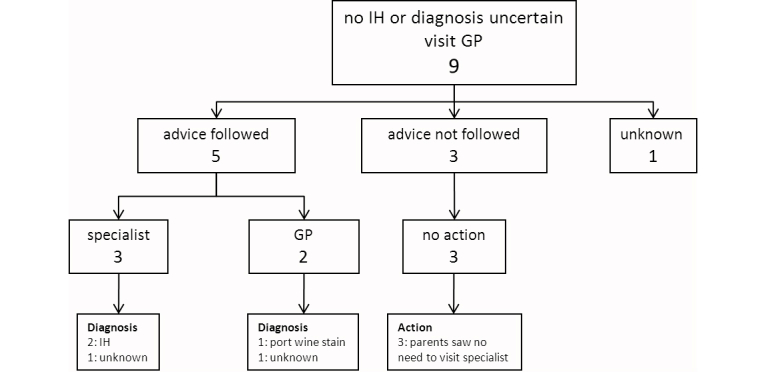
Flowchart of the compliance of the parents of a child with no Infantile hemangioma (IH) or where it was not possible to make an accurate diagnosis by the dermatologist via e-consultation. The flowchart shows which doctor the parents visited and to what actions (eg, diagnostic evaluation and/or treatment) it has led. The figures indicate the number of patients. General practitioner (GP).

There were 119/128 (93.0%) skin lesions that were diagnosed as an IH of which 58/119 (48.7%) parents were advised not to visit the medical specialist, and 61/119 (51.3%) parents were advised to visit a medical specialist. In 9/119 cases (7.6%) the skin lesion was not an IH or the diagnosis was uncertain. A total of 110/128 (85.9%) parents followed the advice of the dermatologist. Figure 2 shows all patients who were advised not to visit a medical specialist. Figure 3 shows all patients, who were advised to visit a medical specialist. Figure 4 shows all patients with no IH or where it was not possible to make an accurate diagnosis.

There were 8/58 parents who were advised not to visit a specialist that did visit a medical specialist (for unknown reasons) (Figure 2). In four patients beta blocker treatment was initiated–one patient with a small, superficial, localized/nodular IH in the face was treated with oral propranolol; one patient with a superficial, localized/nodular IH on the lower arm was treated with topical timolol; and two patients with a small, superficial, localized/nodular IH in the face/neck area were treated with topical timolol. There were 3/61 parents who were advised to visit a specialist and did not–one small, superficial, localized/nodular IH close to the eye spontaneously went into regression; and two parents saw no need to visit a specialist (one patient with a big superficial IH on the arm, because of no functional impairment, and one patient with a small superficial IH on the tip of the nose whose parents did not want treatment). In three cases of “no IH/uncertain diagnosis” the advice of the dermatologist was not followed because the parents saw no need to visit a specialist (Figure 4).

The time between the advice and the actual appointment with a medical specialist, sorted by referral indication, are shown in Table 3. These data were available for 33/71 cases.

**Table 3:** Compliance, time between the advice and the actual appointment with a specialist, and average age of the patient, sorted by referral indication.

Referral indication	n^a^	Compliance, n (%)	Average time to appointment in weeks, (SD^b^)	Average age of the child weeks, (SD)
(Imminent) functional impairment	19	18 (94.7)	2.6 (2.5)	12.6 (9.1)
Ulceration	19	18 (94.7)	3.4 (2.6)	13.5 (9.4)
Cosmetic impairment	18	17 (94.4)	3.9 (5.0)	55.5 (143.4^c^)
Diagnostic	15	14 (93.3)	2.8 (3.1)	9.3 (5.3)

^a^n=number of patients

^b^Based on available data in 33/71 cases.

^c^One patient with cosmetic impairment was 12 years. Excluding this patient the average age was 22.1 SD 21.8 weeks.

### Acceptance and Usability

On all questions concerning PU an average of 91.3% (116.8/128) (range 86.7%, 111/128-98.5%, 126/128) of the parents agreed. This means that the PU was high.

Almost all parents (98.4%, 126/128) found the information of the e-learning understandable and clear, and 92.2% (118/128) of them found the eHealth intervention easy to use. There were 3/128 parents (2.3%) that experienced technical problems with logging in, 3/128 (2.3%) with filling in the questionnaire, and 29/128 parents (22.6%) experienced technical problems with uploading the photograph of their child.

The majority of the parents (94.8%, 121/128) found the eHealth intervention reliable and most of them (98.4%, 126/128) would recommend the eHealth intervention to other parents. There were 97.7% (125/128) of them that think the time investment was worth the effort (average time of completing the e-learning module, excluding e-consult, was 12.54 minutes). The average rate parents gave the eHealth intervention on a 0-10 scale was 8.4 (SD 1.1).

Comments and suggestions were evaluated. Positive comments were given about the reassurance parents experienced, the added value of the e-learning module for primary health care providers, and timely and adequate care due to the eHealth intervention. Negative comments were given about “shocking” photographs used in the e-learning module and difficulties in uploading photographs from an iPad.

An evaluation of difference in acceptance, usability, and attitude between compliant parents and noncompliant parents showed that noncompliant parents judged the eHealth intervention significantly less reliable compared to the compliant parents (71.4%, 10/14 versus 97.3%, 107/110), *P*=.003). There was no statistically significant difference between the percentage of highly educated parents in the compliant group (68.2%, 75/110) and the noncompliant group (85.7%, 12/14) (*P*=.23). All parents with a low education level (n=6) found the eHealth intervention easy to use.

## Discussion

### Parent Compliance With Advice

This study shows that parents are highly compliant (85.9%, 110/128) to the advice of the dermatologist given via the described eHealth intervention for IH. Overall parents very positively judged the PU and PEU and they had a positive attitude towards the eHealth intervention.

The compliance rate is high compared to patient compliance with telephone triage recommendations in emergency care (62%), compliance to advice given via Web-based triage in primary care (57%), and family compliance to travel advice (≥80%) [31-33]. The high compliance of the eHealth intervention might have been positively influenced by its perceived reliability. Our eHealth intervention addresses the need of parents to get complementary information regarding diagnosis and treatment, to get a second opinion, to complement the information already provided by their doctor, or to confirm what they are already thinking [8,34]. It was developed in cooperation with the HEVAS and parents could find it by means of a link on their home page [21]. On the home page of the open access eHealth intervention the logos of HEVAS and UMCU were shown, as well as the names of the specialists of the CAVU team. All this might have contributed to the reliability of our eHealth intervention and might have increased the compliance of the parents. This is confirmed by the fact that noncompliant parents judged the eHealth intervention significantly less reliable.

Little is known about (non) compliance to advice given via eHealth. Compliance is a multifaceted process that is influenced by multiple factors (eg, social and economic circumstances, particularly health literacy, patient belief systems and patient education) [35,36]. Noncompliance to the advice may reflect ignorance or misunderstanding of the clinical situation and might result from the parents’ inability to cope emotionally with the stresses surrounding the advice [37]. The advice, given by e-consultation, might have been in conflict with previously obtained advice by the parents from, for example, other health care takers, family, friends, media sources and health-related websites. Parents who encountered conflicting information might have been less compliant to the advice [38]. Principles to improve compliance to medication have been described and mostly apply in the case of a face-to-face contact between doctor and patient/parent [35]. Therefore most of these principles do not apply to compliance to the advice given via our eHealth intervention. Further studies are necessary to evaluate the factors influencing (non) compliance to advice given via eHealth.

The advice given via the eHealth intervention was based on criteria used in the literature [4,39-41] and in line with a recently published consensus about the treatment of IH with propranolol [6]. However, treatment was initiated in four children who were advised not to visit a medical specialist (Figure 2) and in 15 children visiting a GP/medical specialist has not led to action (Figure 3). A possible explanation is that in some cases our advice was inadequate because of the lack of information given by the parents (eg, photograph of the IH did not reflect the real situation). Another explanation might be that not all GPs and medical specialists are familiar with the most recent recommendations for the management of IH.

### Parental Education Levels and the Internet

In accordance with findings about parental Internet use for health-related information in the literature, the population of this study consisted of highly educated woman in the age group 30-35 [10,42,43]. This higher education is associated via higher eHealth literacy [9,44]. Possibly, low educated parents did not find the eHealth intervention on the Internet or dropped out of the e-learning module before finishing because they were not able to locate, evaluate, integrate, and apply the medical information (low eHealth literacy) [44], or had other needs and/or expectations. The small number of low educated parents in this study thought the eHealth intervention was easy to use and they were compliant to the advice. Our results show no significant difference in results between (the small number of) low educated and highly educated parents. Parents with a low socioeconomic status have access to the Internet and their Internet use is high [9,42,45]. The pressure to use the Internet to empower patients and exchange information is increasing and therefore the Internet might still provide an opportunity to reach low educated parents and may prompt them to consult their doctor [9,45]. Eventually this might contribute to timely presentation of high-risk IH, also for children of low educated parents.

Chang et al showed that the mean age of the first visit of IH patients to a specialist is 5 months [39]. The average age at the time of referral of IH leading to functional impairment (12.6 weeks) and the average time to appointment (2.6 weeks) suggest that this eHealth intervention might contribute to earlier presentation of patients with high-risk IH in specialized centers. More studies are necessary to confirm this.

### eHealth Intervention Positively Judged by Parents

The parents positively judged the acceptance and usability of the eHealth intervention. A positive attitude leads to intentions to follow the advice [33], and this might have influenced the compliance to the advice in our study. Although 71.8% (92/128) of the parents (Table 2) previously received information via Internet and/or from their primary health care provider/medical specialist, this eHealth intervention seems to have added value. However, there is still progress to be made. Almost a quarter (22.7%, 29/128) of the parents experienced technical problems with uploading of the photograph. Mostly, because uploading via a tablet was not supported by our website. This problem was temporally solved by giving parents the opportunity to send the photograph via email and is now completely resolved. Furthermore, parents commented on the lack of knowledge among primary health care providers. Initially, we have developed the eHealth intervention for both parents and health care providers. Until now, mostly parents participated in the eHealth intervention. To stimulate usage among health care providers, the link to our eHealth intervention has been since 2013 included in the IH guideline for youth health care providers in the Netherlands. It might be interesting to investigate whether this will improve the usage by health care providers.

### Conclusions

Parents of children with an IH show a high compliance (85.9, 110/128) to the advice (about risk of complications and need to be seen by a medical specialist) given by the dermatologist via the described Web-based eHealth intervention. This high compliance might be positively influenced by the good acceptance and usability of the eHealth intervention. Our results implicate that increasing parents’ knowledge and involving them in the care for IH might result in timely presentation and treatment of children with high-risk IH in specialized centers.

## Abbreviations

CAVU: Center for Congenital Vascular Anomalies Utrecht
GP: general practitioner
HEVAS: Dutch patient support group for Hemangiomas and Vascular Anomalies
IH: infantile hemangiomas
PEU: perceived ease of use
PU: perceived usefulness
TAM: Technology Acceptance Model
UMCU: University Medical Center Utrecht
